# An Antibody Biomarker Associated with *Onchocerca volvulus* Microfilariae Identified by Proteomic Analysis of Parasite Tissues Isolated from Paraffin Embedded *O. volvulus* Nodules by Laser Capture Microdissection

**DOI:** 10.4269/ajtmh.24-0793

**Published:** 2025-06-03

**Authors:** Sarah E. Greene, Kerstin Fischer, Lucia S. Di Maggio, Bruce A. Rosa, Yuefang Huang, Irina Diekmann, Byoung-Kyu Cho, Jessica Lukowski, Young A. Goo, Makedonka Mitreva, Nicholas Opoku, Gary J. Weil, Peter U. Fischer

**Affiliations:** ^1^ Infectious Diseases Division, Department of Pediatrics, School of Medicine, Washington University, St Louis, Missouri;; ^2^ Infectious Diseases Division, Department of Medicine, School of Medicine, Washington University, St Louis, Missouri;; ^3^ Mass Spectrometry Technology Access Center at McDonnell Genome Institute, School of Medicine, Washington University, St. Louis, Missouri;; ^4^ McDonnell Genome Institute, School of Medicine, Washington University, St Louis, Missouri;; ^5^ Department of Genetics, School of Medicine, Washington University, St Louis, Missouri;; ^6^ Fred Newton Binka School of Public Health, University of Health and Allied Sciences, Ho, Ghana

## Abstract

Onchocerciasis (river blindness), a neglected tropical disease caused by the filarial nematode *Onchocerca* *volvulus*, impacts millions of people in sub-Saharan Africa. The WHO coordinates global efforts to eliminate onchocerciasis and has prioritized development of improved diagnostic tests to aid these efforts. To find new microfilarial-associated diagnostic targets to help identify active infections, we used laser capture microdissection to isolate embryonic stages from histologic sections of *O. volvulus* worms in subcutaneous nodules excised from onchocerciasis patients. Proteomic analysis identified 2,512 *O. volvulus* proteins in those tissues, including 264 found only in the microfilariae (Mf). From this pool of diagnostic candidates, we selected OVOC12404, a putative cuticular collagen, for further study because of its abundance and lack of close homologues in other filarial species. Immunolocalization detected this antigen on the surface of coiled, stretched, and tissue Mf. IgG antibodies to OVOC12404 were detected by ELISA in plasma from 124 of 162 (76.5%) people with *O. volvulus* Mf in their skin snips. ELISA specificity was 98% based on 42 samples from lymphatic filariasis patients from areas without co-endemic onchocerciasis. In contrast to antibodies to Ov16, a currently used diagnostic target, antibodies to OVOC12404 declined significantly after treatments that cleared *O. volvulus* Mf from the skin. This study showed that proteomic analysis of parasite tissues recovered from histological sections can be used to identify stage-specific filarial diagnostic targets. Further studies are needed to assess the potential value of an OVOC12404 antibody test as an additional diagnostic tool to support the onchocerciasis elimination efforts.

## INTRODUCTION

Onchocerciasis, or “river blindness,” is a neglected tropical disease that is the world’s second leading infectious cause of blindness. It is estimated that approximately 15 million people are infected and at least 249.5 million are at risk of infection in 28 countries, mostly in sub-Saharan Africa.^1^^,^^2^

Onchocerciasis is caused by infection with the filarial worm *Onchocerca* *volvulus* that is spread by the bite of *Simulium* black flies.^3^ These flies transmit infective larvae that can develop into large adult worms in humans over 6 to 12 months. The adult worms live in subcutaneous nodules for up to 15 years. Adult male and female worms mate during reproductive periods, then a female worm can release up to 1,500 first-stage larvae called microfilariae (Mf) per day.^3^ Mf migrate from nodules into the skin then black flies ingest Mf when they bite an infected host. Mf develop in the black fly into infectious L3 larvae that can infect another person. Host responses to Mf in the eyes and skin lead to clinical manifestations of onchocerciasis, including vision loss, severe dermatitis, disabling pruritus, or stigmatizing loss of skin pigmentation and elasticity.

Onchocerciasis is treated with ivermectin, which clears Mf and temporarily sterilizes adult worms. The WHO coordinates efforts to eliminate onchocerciasis with mass drug administration (MDA) of ivermectin. This global program treated 172 million people in 2023, which was 69% of the total population estimated to require MDA.^2^ The elimination program is hampered by a lack of convenient diagnostic tests for detecting active onchocerciasis with Mf present in nodules or in skin. There are many proposed antibody-based diagnostic tests for onchocerciasis with varying sensitivities and specificities.^4^ The most commonly used serologic tests detect IgG4 antibodies to Ov16. Commercially available tests for Ov16 are reasonably sensitive for *O. volvulus* infection but not specific for active infections.^5^^,^^6^ The WHO includes detection of antibodies to Ov16 as part of the decision-making guidelines for stopping MDA.^7^ However, the WHO has also identified the development of improved diagnostic tests as a priority for the onchocerciasis elimination program, both to aid in infection mapping and for MDA stopping decisions.^8^ Other studies have described additional onchocerciasis biomarkers to be used alone or in conjunction with Ov16 to increase sensitivity and/or specificity. These include OV10, OV103, OV33, OVOC3261, Ovcol-1, multiplexed peptides (OvMP-1, OvMP-23, and OvNMP48), and OvMANE1.^4^^,^^9^^–^^13^ One limitation of these biplex tests is that antibodies to Ov16 persist for many years after infections have cleared, although not indefinitely.^14^^–^^17^ Furthermore, several of these diagnostic targets also have issues with sensitivity or specificity, and none of these are used in commercially available diagnostic tests.^4^^,^^18^ Onchocerciasis can also be diagnosed by detecting Mf in skin biopsies (skin snips) using microscopy or polymerase chain reaction. However, skin-snip testing is often not well accepted by local communities, especially when disease burdens are low. Skin-snip testing is also insensitive for detecting early infections (before Mf reach the skin), infected people recently treated with ivermectin, or infected people with low skin Mf counts. We set out to make an improved onchocerciasis diagnostic test to identify the presence of viable and fertile adult female worms or Mf, which are essential for transmission by black flies.

In this study we used laser capture microdissection on ethanol preserved, paraffin-embedded sections of *O.* nodules followed by highly sensitive mass spectrometry to identify new diagnostic candidates. This allowed us to identify proteins present in specific parasite tissues and stages, including adult female worm tissue, ovary, morula, and stretched or mature Mf. We selected OVOC12404 for further study based on its abundance in Mf and the lack of highly homologous proteins in other filarial species. We found that detection of antibodies to OVOC12404 by ELISA is sensitive and specific for onchocerciasis and that antibodies to this antigen decline significantly after treatment that clears Mf from the skin. Therefore, detection of antibodies to OVOC12404 in patient blood could be a useful tool to support the global campaign to eliminate onchocerciasis.

## MATERIALS AND METHODS

### Ethics.

The protocol for the trial to collect onchocerciasis nodules was reviewed by the ethical review committees of University of Health and Allies Sciences (Ho, Ghana), the Ghana Health Service, the Ghana Food and Drug Administration, Case-Western Reserve University (Cleveland, OH), and Washington University School of Medicine in St. Louis (St. Louis, MO), institutional review board [IRB] identification: 201910085.^19^ We used de-identified sera and plasma samples from individuals with various parasitic infections. We also used de-identified non-endemic control plasma samples from the Barnes-Jewish Hospital clinical laboratory in St. Louis, Missouri. The Washington University in St. Louis Human Research Protection office (an IRB) determined that use of these de-identified samples did not constitute human subjects research (IRB# 202308091).

### Onchocerciasis nodules and human plasma samples.

We used de-identified legacy *O. volvulus* nodules that were surgically removed as part of a clinical trial in Ghana, then ethanol fixed and paraffin embedded.^19^ We also used de-identified legacy *O. volvulus* nodules from untreated onchocerciasis patients from Liberia.^20^ These nodules were formalin fixed and paraffin embedded. We also used archived de-identified plasma samples from individuals infected with filarial parasites as specified in Table 1.

**Table 1:** Plasma sample characteristics

Infection Status	Country of Collection	Number Tested	Diagnosis Method	Coinfections	Ref
*O. volvulus*	Uganda	70	Mf count from skin snip	*M. perstans *possible	^21^
	Ghana	92	Mf count from skin snip		^19^
*W. bancrofti*	Côte d’Ivoire	21	Night blood Mf count		^22^
	Egypt	4	Night blood Mf count	Absent	^23^
	India	17	Night blood Mf count	Absent	^24^
	Sri Lanka	21	Night blood Mf count	Absent	^25^
*M. perstans*	Uganda	16	Day blood Mf count	Potentially *O. volvulus* exposed	^26^
*L. loa*	Cameroon	27	Day blood Mf count	Potentially *O. volvulus* exposed	^27^ ^, ^ ^28^
Non-endemic control	United States	16	Not applicable	Absent*	^29^

*L.* = *Loa*; *M. *= *Mansonella*; Mf = microfilariae; O. = *Onchocerca*;* W. *= *Wuchereria*; Ref, reference.

* Non-endemic control plasma from the United States is presumed to be free from filarial infections based on local epidemiology and travel patterns.

### Laser capture microdissection and mass spectrometry.

Histologic sections from three nodules removed from three different people with onchocerciasis were used.^19^ Tissues from different developmental stages were identified and used for laser capture microdissection (LCM) as previously described.^30^ We collected oocytes, morula, and stretched Mf from within the uterus of adult female worms. We also collected body wall tissue to represent adult females. Briefly, collected LCM tissues were lysed by heat and sonicated in 5% sodium deoxycholate. Samples were reduced with 4 mM dithiothreitol, and the cysteine residues were alkylated with 18 mM iodoacetamide in the dark. Proteins were then trypsin digested. Sample peptides were desalted using solid-phase extraction with a C18 spin column and eluted with 0.1% trifluoroacetic acid in 50% acetonitrile, then reconstituted in 0.1% formic acid. Digested peptides were analyzed by liquid chromatography-tandem mass spectrometry (LC-MS/MS) using a Vanquish Neo UHPLC system coupled to an Orbitrap Eclipse Tribrid Mass Spectrometer with a FAIMS Pro Duo interface (Thermo Fisher Scientific, San Jose, CA). MS acquisition was conducted as previously described.^30^ The mass spectrometry proteomics data have been deposited to the ProteomeXchange Consortium via the PRIDE partner repository with the dataset accession number PDX057556 and digital object identifier 10.6019/PXD057556.^31^

### Bioinformatic analysis of proteomics data.

Data were analyzed as previously described.^32^^,^^33^ Identified peptides were compared with *O. volvulus* and human (common Repository of Adventitious Proteins) peptide databases. Peptides found in the host database were removed from the analysis. We used Scaffold version 5.2.2 to analyze the data (Proteome Software Inc., Portland, OR). We included proteins with a 95% peptide threshold and a <1% false discovery rate. The normalized spectral abundance factor (NSAF) was used to quantify and prioritize the proteins. Proteins were included if they were identified by the presence of least two peptides per protein and if they were detected in at least two of three biological replicates. The spectra and peptide count results for the included proteins are available, with proteins ranked by NSAF (Supplemental Table 1). Potentially secreted proteins were identified using signal peptide sequences identified by SignalP v 5.0 as previously described.^33^ Protein conservation across nematodes and other hosts was also assessed using Orthofinder v2.4.1 as previously described.^32^^,^^33^ Briefly, worm protein sets were downloaded from WormBase Parasite version WBPS15 WS276^34^ (https://parasite.wormbase.org) for *Ancylostoma ceylanicum* (PRJNA72583), *Loa loa* (PRJNA246086), *Necator americanus *(PRJNA72135), *Ascaris lumbricoides* (PRJEB4950), *Caenorhabditis elegans* (PRJNA13758), *Brugia malayi* (PRJNA10729), *Trichuris trichiura* (PRJEB535), *Strongyloides stercoralis* (PRJEB528), *O. ochengi* (PRJEB1465) and *Wuchereria bancrofti* (PRJNA275548, with annotation improvements made). Host outgroup proteins were downloaded from Ensembl^35^ for *Oryctolagus cuniculus* (Rabbit; OryCun2.0), *Homo sapiens* (Human; GRCh38.p13), and *Drosophila melanogaster *(BDGP6.32). OrthoFinder used phylogenetic analyses to identify homologous proteins and group them into orthologous protein families (OPFs), and orthology was defined according to the presence or absence of orthologs from other species in the OPF for each *O. volvulus* protein.^36^ We also searched for proteins with high sequence similarity to OVOC12404 using BASIC Local Alignment Search Tool (BLAST) in the National Center for Biotechnology Information (https://blast.ncbi.nlm.nih.gov/Blast.cgi) and WormBaseParasite databases. The identified collagen proteins from *O. volvulus, Loa loa, Wuchereria bancrofti, Brugia malayi, *and *Dirofilaria immitis* were aligned with Clustal W in MegAlign Pro version 17 (DNAStar, Madison, WI). A bootstrap consensus tree of these proteins was constructed in MEGA11. This tree was inferred from 1,000 replicates using the maximum likelihood method.^37^

### Protein production.

We were unable to amplify the *O. volvulus* gene encoding OVOC12404 from *O. volvulus* complementary DNA, so we purchased double-stranded DNA synthesized by Integrated DNA Technologies (Coralville, IA), based on available sequence for this gene in WormBaseParasite.^34^ We were unable to produce protein in *Escherichia coli* using expression vectors that usually work to express filarial proteins. Therefore, we had the protein of interest OVOC12404 chemically synthesized (GenicBio Limited, Shanghai, China). Ov16-GST fusion protein was produced as previously described.^38^

### Antibody production.

Polyclonal rabbit antibodies were commercially raised against synthesized OVOC12404 and purified by protein A affinity chromatography (LifeTein, Somerset, NJ).

### Localization of OVOC12404 in *O. volvulus*.

For immunohistochemistry, we used histologic sections of six different nodules that were removed and fixed as described above.^19^^,^^20^ These sections were stained using the alkaline phosphatase anti-alkaline phosphatase method as previously described.^29^^,^^39^ Rabbit polyclonal antisera raised against OVOC12404 was used as the primary antibody at a dilution of 1:300. Preimmunization sera from the same rabbit was used as the negative control, also at a dilution of 1:300. The slides were examined on an Olympus-BX40 microscope and photographed on an Olympus DP70 microscope digital camera (Olympus, Tokyo, Japan). We also used confocal laser scanning microscopy as previously described.^40^ Briefly, we used Alexa-488 conjugated anti-rabbit IgG at 1:200 (Sigma, St Louis, Mo) as the secondary antibody, Wheat Germ Agglutinin 633 (Thermo Fisher Scientific) at 1:5 for membrane labeling, and ProLong^TM ^Gold antifade reagent with DAPI (Thermo Fisher Scientific) as mounting medium. Sections were examined on a Zeiss LSM T-PMT (Zeiss, Jena, Germany). Confocal Z slices of 0.8 µm were obtained with Zeiss LSM software (ZEN). Confocal laser scanning microscopy was performed at the Washington University Molecular Microbiology Imaging facility (https://sites.wustl.edu/imaging/).

### Detection of anti-OVOC12404 antibodies in human plasma by ELISA.

We coated 96 well round bottom ELISA plates (Immunlon 2 HB, ThermoFischer Scientific, Waltham, MA) with 100 µL of OVOC12404 antigen diluted to 2 µg/ml in 0.06 M carbonate buffer pH 9.6 and incubated the plates overnight at 37°C in a humidified chamber. Plates were washed five times in phosphate buffered saline (PBS) with 0.05% Tween (PBST) and then blocked in PBST with 5% heat inactivated fetal calf sera (ELISA diluent) at 37°C for 2 hours. Human plasma was diluted to 1:300 in ELISA diluent and 100 µL was added to duplicate wells. Plates were incubated at 37°C for 2 hours, then washed five times with PBST. We added 100 µL per well of anti-human IgG-conjugated to horseradish peroxidase (Southern Biotech, Birmingham, AL) diluted to 1:4000 in ELISA diluent for 1 hour at 37°C. Plates were washed in PBST five times. We used the colorimetric substrate o-phenylenediamine dihydrochloride (Thermo Fisher Scientific), per manufacturer’s instructions. The enzymatic reaction was stopped with 50 µL per well of 4 M sulfuric acid and plates were read at 490 nm with a BioTek ELx808 plate reader (Thermo Fisher Scientific). Optical density values from duplicate wells were averaged. An optical density positive cutoff of 0.2 was chosen based on results of a receiver operating curve analysis (Supplemental Figure 1). Data were analyzed with Excel software (Microsoft, Redmond, WA) and Prism version 9 (GraphPad, San Diego, CA).

### Detection of anti-Ov16 antibodies in human plasma by ELISA.

We coated 96 well round bottom ELISA plates (Immunlon 2 HB, ThermoFischer Scientific, Waltham, MA) with 100 µL of Ov16-GST antigen diluted to 2 µg/ml in 0.06 M carbonate buffer pH 9.6 and incubated the plates overnight at 37°C in a humidified chamber. Plates were washed five times in PBST, and then blocked in ELISA diluent at 37°C for 2 hours. Human plasma was diluted to 1:100 in ELISA diluent and 100 µL was added to duplicate wells. Plates were incubated at 37°C for 2 hours, then washed five times with PBST. We added 100 µL per well of anti-human IgG4-Fc’ conjugated to horseradish peroxidase (Southern Biotech, Birmingham, AL) diluted to 1:4000 in ELISA diluent for 1 hour at 37°C. Plates were washed in PBST five times. We used a colorimetric readout and analyzed the data, as described for the anti-OVOC12404 ELISA above. An optical density positive cutoff of 0.2 was used.

## RESULTS

### Proteomic analysis of stage specific *O. volvulus* tissue.

**Figure 1.**
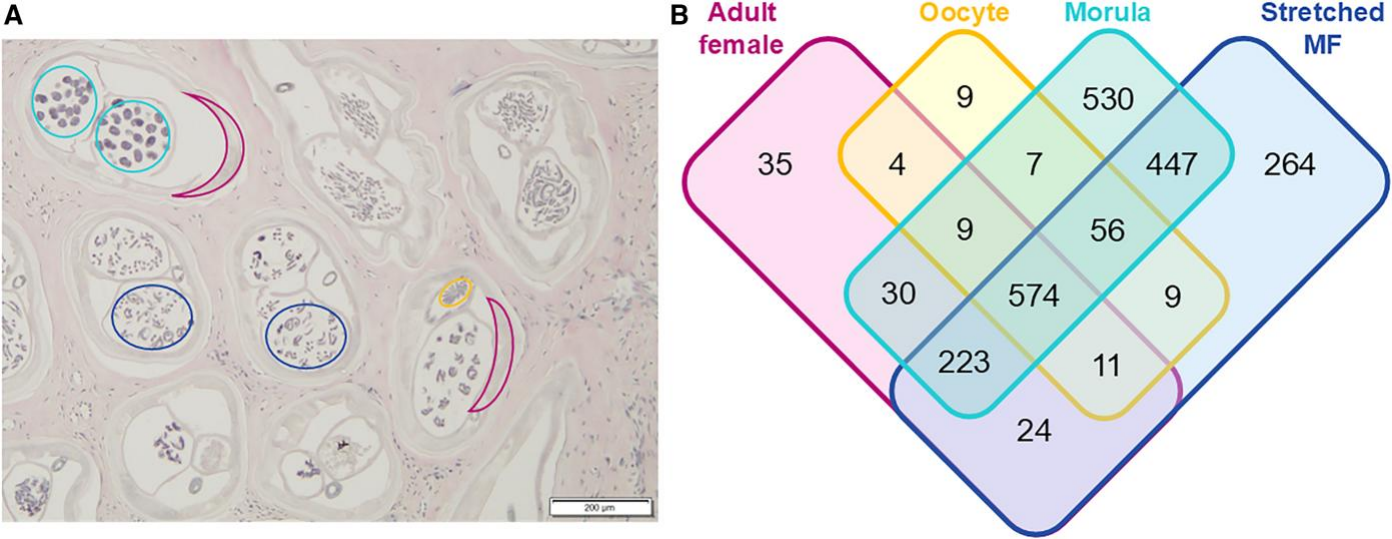
Laser capture microdissection. (**A**) Histologic section of an *O. volvulus* nodule demonstrating examples of the tissues collected: adult female tissue in purple, oocyte in yellow, morula in teal, and microfilariae (Mf) in blue. (**B**) Venn diagram of the number of proteins identified in each *O. volvulus *worm tissue. Overlapping areas demonstrate the number of proteins found in the indicated compartments.

**Figure 2.**
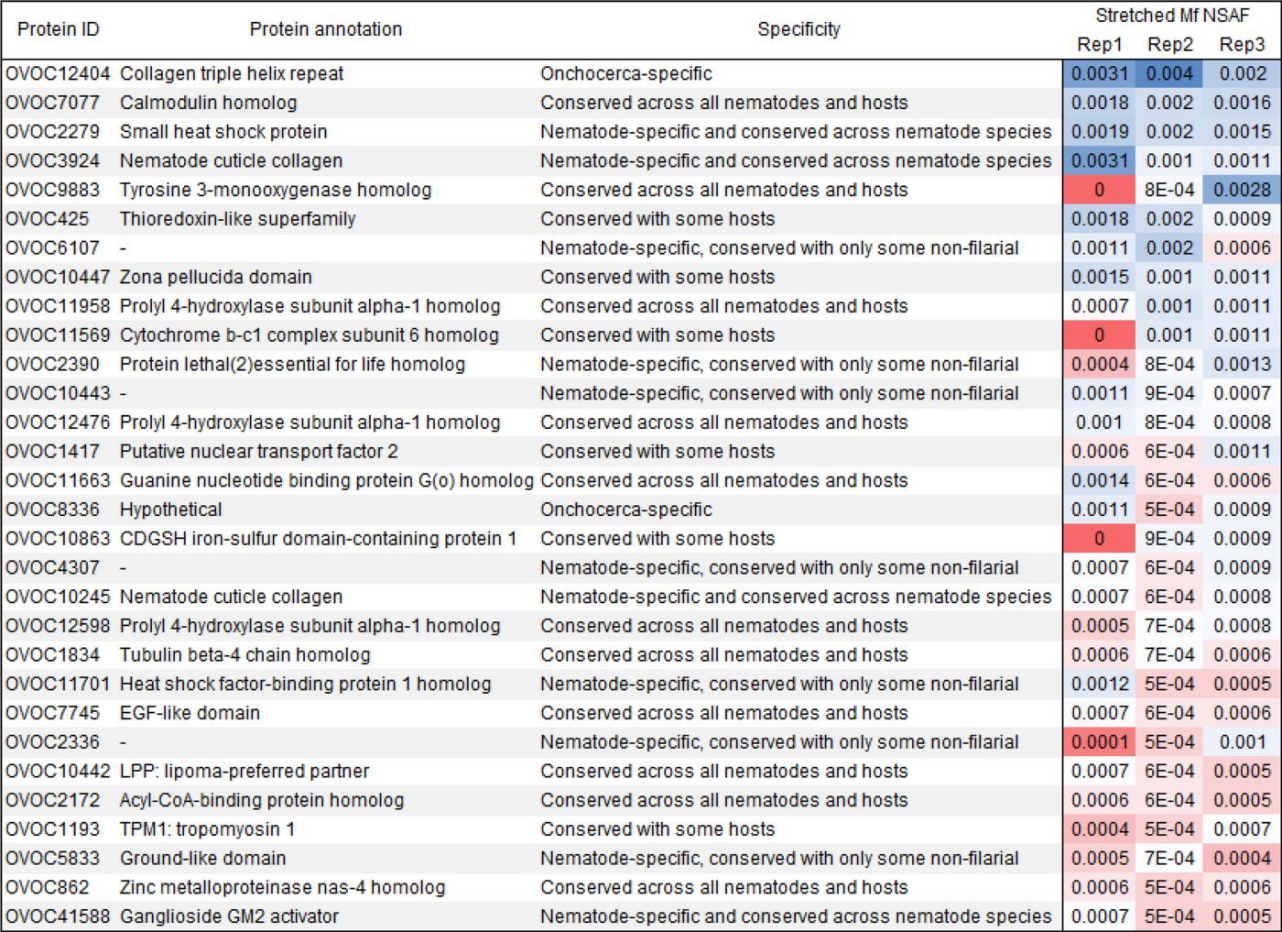
Top 25 most abundant proteins only identified in microfilaria. Normalized spectral abundance factor (NSAF) values for each of the three biological replicates are shown. Colors indicate relative abundance, with blue indicating higher abundance and red indicating lower abundance. Proteins are ranked based on the average NSAF for all three biological replicates, with the most abundant proteins at the top of the list.

We used highly sensitive mass spectrometry to identify proteins in adult female worms within three subcutaneous nodules that had been surgically removed from three infected humans. We used laser capture microdissection of histologic sections of nodules to perform detailed spatial proteomic analyses on adult female worm tissues and developmental stages including ovary, morula, and stretched Mf (Figure 1A). The proteomic analysis of worm tissues isolated by laser capture microdissection identified 2,512 *O. volvulus* proteins that met the inclusion criteria, as detailed in the Methods section (Supplemental Table 1). Many of these proteins were present in multiple developmental stages. However, 838 proteins were only identified in one tissue type alone (Figure 1B). These included 264 proteins only detected in stretched Mf. We focused on Mf-specific proteins because we were interested in developing a diagnostic test that correlated with infections with ongoing Mf production. We ranked the 264 Mf-specific proteins by average NSAF, and the 25 most abundant of these included many proteins that are highly conserved across different filarial species (Figure 2). We considered these proteins unlikely to lead to the development of a specific diagnostic test. However, two Mf-specific proteins, OVOC12404 and OVOC8336, do not have close homologues in other filarial species, based on a phylogenetic analysis to identify homologues in Orthofinder (Supplemental Table 1). OVOC12404 was the most abundant of the Mf-specific proteins. It is one of only four Mf-specific proteins identified in all three of the nodules analyzed. OVOC12404 is a small, 98-amino acid protein, with a predicted molecular weight of 9.6 kilo daltons. This protein includes a signal peptide, making it likely to be secreted (Supplemental Table 1). Based on homology with other proteins and the Gly-X-Y peptide triplets in the C-terminus of the protein, it is predicted that OVOC12404 is a collagen with a collagen triple helix. Therefore, we hypothesized OVOC12404 might be a component of the Mf cuticle and potentially involved in host-pathogen interactions. We further characterized OVOC12404 as a potential candidate for immunodiagnosis. We used BLAST to identify similar proteins in other filarial species and identified collagen proteins in these organisms with 46–68% amino acid similarity to OVOC12404 (Supplemental Figure 2). These included another *O. volvulus* collagen, Ovcol-1, and an *O. volvulus* collagen-like protein that were both previously described.^11^ We constructed an amino acid alignment of some of these filarial collagens (Supplemental Figure 2), as well as a phylogenetic tree (Supplemental Figure 3). This tree demonstrated that the collagen most closely related to OVOC12404 is OVOC12491, which also had the highest percent similarity to OVOC12404.

### Immunohistochemical localization of OVOC12404.

**Figure 3.**
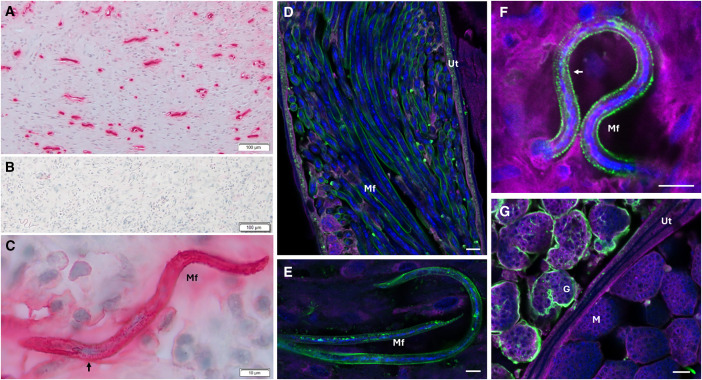
Immunohistochemical localization of OVOC12404. Images demonstrate immunohistochemical (IHC) done by alkaline phosphatase anti-alkaline phosphatase (APAAP) (**A**,** B**, and** C**) or confocal laser scanning microscopy (**D**, **E**, **F**, and **G**). Shown are *O. volvulus* nodule sections stained with anti-OVOC12404 rabbit antibodies (**A**) or pre-immune sera from the same rabbit (**B**). (**C**) A higher magnification image demonstrating a free microfilariae (Mf) within the nodule stained with anti-OVOC12404. **(D**) Longitudinal and cross-sectional image of Mf within the uterus of an adult female worm. Anti-OVOC12404 staining of the surface of Mf (**E** and** F**) and the gastrula but not the morula stage (**G**). For the confocal laser scanning microscopy in panels **D**–**G**: anti-OVOC12404 is shown in green, DAPI (DNA stain) is shown in blue, wheat-germ agglutinin 633 (membrane stain) is shown in magenta, and white scale bars indicate 10 *µ*m. (**C** and** F**) Arrows indicate striations on the Mf surface. G = gastrula; M = morula; Ut = uterus.

We conducted immunohistochemical localization to characterize OVOC12404 within onchocerciasis nodules. We analyzed histologic sections from six different nodules, and representative images from two nodules are shown in Figure 3A–C. Polyclonal rabbit antibody to OVOC12404 strongly labeled coiled and stretched Mf. We saw a banding pattern consistent with annular striations along the Mf surface (Figure 3C), possibly indicating that this protein is part of the Mf cuticle. No strong labeling was seen at the cuticle of the adult female worm. Interestingly, there was also a halo of staining around the free Mf in the nodule, which we attributed to secreted or shed OVOC12404 outside the Mf, possibly marking the track of their migration within the nodule. We conducted confocal laser-scanning microscopy on nodules collected in the same study as the nodules initially used for the laser capture microdissection (Figure 3D and G), as well as nodules collected from untreated onchocerciasis patients in Liberia (Figure 3E and F). We saw similar patterns of localization in nodules from both locations. Using confocal microscopy, it was clear that anti-OVOC12404 antibodies bound to the outer surface of the Mf, both those free in the nodule and those within the uterus of the female. We again saw the banding pattern consistent with striations along the surface (Figure 3F). Interestingly, anti-OVOC12404 antibodies did not bind to the morula stage of the developing larvae, but did bind to the gastrula, a stage later than morula but before the larvae develop into stretched Mf (Figure 3G).

### Detection of anti-OVOC12404 antibodies in human plasma.

**Figure 4.**
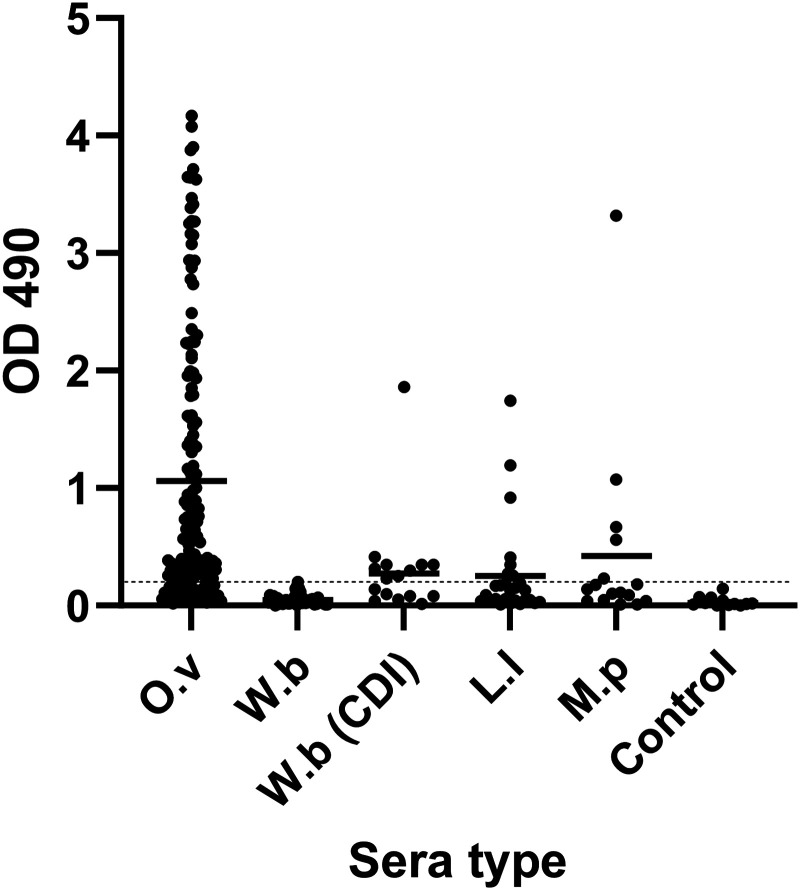
Anti-OVOC12404 IgG ELISA results for plasma from people with the indicated filarial infection. Graph shows the individual OD_490_ values for the anti-OVOC12404 IgG ELISA from people with the indicated filarial infection: *Onchocerca volvulus* (O. v), *Wuchereria bancrofti* (W. b), *Loa loa* (L. l), or *Mansonella perstans* (M. p). Median values are indicated by the black bar. The positivity cutoff of 0.2 is indicated by the dotted line.

We used two methods to assess the potential utility of detecting antibodies to OVOC12404 for diagnosis of active *O. volvulus* infection. Initial studies used immunoblot with synthesized OVOC12404. Most filarial serodiagnostics assess for IgG4 antibodies to filarial antigens; however, only six of 12 (50%) plasma samples from people with onchocerciasis had detectible IgG4 against OVOC12404. Six of eight (75%) plasma samples had IgG antibodies to OVOC12404 and no plasma samples from nine people without onchocerciasis had anti-OVOC12404 IgG detectible by immunoblot. Therefore, given improved sensitivity of detecting anti-OVOC12404 IgG over IgG4 without sacrificing specificity, we developed an ELISA for detecting IgG anti-OVOC12404 antibodies in a high throughput manner. With this assay, we tested plasma from people with onchocerciasis and from people infected with related filarial parasites. ELISA optical density data are shown (Figure 4). Although OVOC12404 was predicted to be Mf associated, ELISA results were not significantly correlated with the Mf count from skin snips (Supplemental Figure 4). Assay sensitivity and specificity are shown in Table 2. Assay sensitivity was based on onchocerciasis plasma samples from Uganda and Ghana, and the ELISA results from these two countries were statistically different by Mann-Whitney *U* test (*P* = 0.0059). Interestingly, the Mf counts were also significantly different between the samples from these two countries (Supplemental Figure 5). Because of the overlapping endemicity of some filarial infections, it can be challenging to find samples from people in onchocerciasis endemic areas who have not been exposed to *O. volvulus*. This complicates testing for assay cross reactivity because of *Mansonella perstans* and *Loa loa* infections. We tested plasma from people with *Wuchereria bancrofti* infection, both from Cote d’Ivoire (CDI), where onchocerciasis exposure is possible, and from Egypt, India, and Sri Lanka, where onchocerciasis is not present. The assay specificity based on plasma from CDI was 57%, whereas specificity based on plasma from onchocerciasis unexposed areas was 97.6%. Furthermore, the one positive sample from a *Wuchereria bancrofti*-infected person from Sri Lanka had a low OD_490_ value, just above the positive cutoff of 0.2. The specificity of the assay with non-endemic control plasma was 100% (Table 2).

**Table 2:** Anti-OVOC12404 IgG ELISA sensitivity and specificity

Infection Status	Country	# Tested	# Positive (%)	Sensitivity	Specificity
*O. volvulus*	Uganda	70	49 (70)	76.5	–
	Ghana	92	75 (81.5)		
*W. bancrofti*	CDI	21	9 (43)	–	57
	Egypt	4	0 (0)	–	97.6
	India	17	0 (0)		
	Sri Lanka	21	1 (5)		
*M. perstans*	Uganda	16	5	–	69
*L. loa*	Cameroon	27	7	–	74
Non-endemic control	United States	16	0 (0)	–	100

*L.* = *Loa*; *M. *= *Mansonella*; O. = *Onchocerca*;* W. *= *Wuchereria*.

### Impact of treatment on detection of anti-OVOC12404 antibodies.

**Figure 5.**
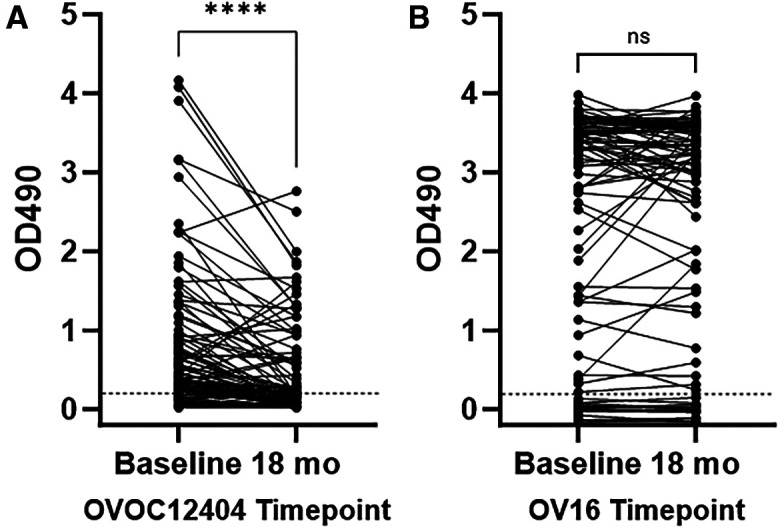
OVOC12404 ELISA before and after treatment. Graph shows the individual OD_490_ values for the anti-OVOC12404 IgG ELISA (**A**) or the anti-Ov16 IgG_4_ ELISA (**B**) before treatment (baseline) and 18 months (18 months) after treatment. Black lines connect the OD_490_ values from each individual at the baseline and 18 months timepoints. The positivity cutoff of 0.2 is indicated by the dotted line. By Wilcoxon matched-pairs sign rank test comparing pre- and posttreatment samples, the median anti-OVOC12404 IgG values decreased significantly, *P* <0.0001 (**A**), but the anti-Ov16 IgG_4_ values were not significantly changed, *P* = 0.1528 (**B**).

Optimally, a new onchocerciasis diagnostic test would have high sensitivity and specificity and be more specific than available tests for detecting infections with living female worms that are producing Mf. To test the impact of treatment on anti-OVOC12404 antibody levels, we used plasma from a treatment trial where onchocerciasis patients were treated with different combinations of drugs active against *O. volvulus*, including ivermectin.^19^ We tested 92 baseline plasma samples from before participants were treated as part of the analysis of assay sensitivity described above. Here, we tested paired plasma samples from these 92 participants obtained 18 months after they were treated. At baseline, 75 (81.5%) of these participants had detectible anti-OVOC12404 IgG. By 18 months after treatment, 63 of these people had decreased ELISA OD_490_ values, with only 44 (48%) having detectible antibody to OVOC12404 (Figure 5A). Twelve people had increased OD_490 _values relative to their baseline by 18 months after treatment, but four of these had only slight increases in their OD_490_ values of <20%. Three people had no detectible anti-OVOC12404 antibodies at baseline but developed low but detectable levels of antibody by 18 months follow-up (all had OD_490_ <0.3). Furthermore, the median OD_490_ values were significantly lower 18 months after treatment compared with baseline values. Of the 92 participants tested, 45 (49%) had detectible Mf counts in their skin 18 months after treatment and 43 (47%) had no detectible Mf (there was no skin-snip data for five participants). There was no statistically significant difference in the percentages of people with detectible antibody or in the median OD_490_ values between those with and without Mf in their skin snips 18 months after treatment.

Currently, onchocerciasis can be detected based on presence of IgG4 antibodies to Ov16, a test with excellent specificity and moderate sensitivity.^6^ We used the same paired plasma samples from baseline and 18 months after treatment to measure anti-Ov16 IgG4 antibodies. We found that 71 of 92 (77%) participants had detectible anti-Ov16 IgG4 at the start of the study and at 18 months after treatment (Figure 5B). Of the 71 participants with detectible anti-Ov16 IgG4, 41 had levels that decreased after treatment but 34 of these people had titers that deceased by <20%. Thirty people had increased OD_490_ values by 18 months, but 18 of these were increased by <20%. Furthermore, the median anti-Ov16 antibody OD_490_ values did not change significantly after treatment. All 21 people with non-detectible anti-Ov16 IgG4 antibodies remained non-detectible at 18 months follow-up.

We also compared each individual’s antibody responses to both antigens. At the baseline timepoint, 62% of people had antibodies to both antigens, whereas 20% had antibodies to only OVOC12404 and 15% had antibodies to only Ov16 (Table 3). At baseline, there was no significant difference in percent positivity between the two tests. However, by 18 months after treatment, significantly more people had antibodies to Ov16 than to OVOC12404 by McNemar’s test (*P* <0.0001). If we use this data to predict the performance of a diagnostic test based on antibodies to either OVOC12404 or Ov16, 89 of 92 (97%) of people would test positive before treatment and 78 of 92 (85%) would test positive 18 months after treatment. The baseline and 18-month percent positivity for a potential combination assay are significantly higher than those of Ov16 alone by McNemar’s test (*P* <0.02).

**Table 3:** Antibodies to OVOC12404 and Ov16 in paired plasma samples before and after treatment of onchocerciasis

Time Point	OV12404 (%)	Ov16 (%)	OV12404+ Ov16+ (%)	OV12404+ Ov16− (%)	OV12404-Ov16+ (%)	OV12404-Ov16− (%)
Baseline	75 of 92 (81.5)	71 of 92 (77)	57 (62)	18 (20)	14 (15)	3 (3)
18 months	44 of 92 (48)	71 of 92 (77)	37 (40)	7 (8)	34 (37)	14 (15)

## DISCUSSION AND CONCLUSION

The elimination of human onchocerciasis campaign uses several tests to identify endemic areas and to assess the success of elimination efforts using mass administration of ivermectin. These include detection of *O. volvulus *DNA in black flies (molecular xenomonitoring), detection of *O. volvulus* Mf in skin snips, and detection of antibodies to Ov16. However, skin snips are somewhat invasive and insensitive for detecting active infections in people with low Mf counts or who have been recently treated with ivermectin. Ov16 antibody-based testing also has sensitivity concerns and does not differentiate current from past infection. The WHO has called for the development of improved diagnostic tests to support the onchocerciasis elimination effort.^8^ The WHO has also created a target product profile (TPP) for onchocerciasis diagnostic tests, with the goal of 99.8% specificity, ≥60% sensitivity for mapping endemic areas, and ≥89% sensitivity for MDA stopping decisions.^41^

This study used laser capture microdissection of fixed histologic sections of onchocerciasis nodules, followed by highly sensitive mass spectrometry to identify the proteins found in adult and different embryonic stages of *O. volvulus* worms. This study demonstrates the usefulness of laser capture-mass spectrometry for identifying proteins present in parasite tissues in fixed histological sections. We investigated an Mf-specific protein, but this method could also be used to identify additional diagnostic targets for different *O. volvulus* life stages or tissues. For example, biomarkers for adult *O. volvulus* worms could be useful given the variable results obtained with nodule palpation. Such a marker could also serve to measure drug treatment efficacy, especially for macrofilaricides under development. This method could provide a way to further investigate what controls filarial reproduction and development, as well as explore the relationship between *O. volvulus* and the endosymbiont *Wolbachia*. This approach could also be used to identify additional diagnostic targets in other filarial infections. We propose that this method could be a valuable avenue for identifying tissue-specific protein abundance for pathogens, especially pathogens for which there are no animal models and in which it is difficult to get in vivo samples.

With this spatial proteomic method, we identified 264 Mf-specific proteins, including OVOC12404, which has no known close homologues outside of *Onchocerca*. Immunohistology confirmed the findings of our proteomic analysis regarding Mf localization of this antigen. We observed no anti-OVOC12404 staining of the early developmental stages such as morula, but saw staining of more developed stages, such as gastrula, pretzel, and stretched Mf.^42^ We demonstrated clear anti-OVOC12404 staining of the Mf within the nodule and the nodule tissue immediately surrounding the Mf, further evidence that this protein was on the surface of the Mf and possibly being shed as Mf move through the nodule stroma. Because OVOC12404 was predicted to contain a collagen triple helix and was associated with Mf, we hypothesized the protein was likely present in the Mf sheath around developing Mf or in the cuticle of mature Mf. Some nematode cuticle proteins are specific to particular developmental stages. The cuticle of *O. volvulus* is an interesting organelle that is important for both worm structure and host–pathogen interactions. There has been some research about the cuticle of *O. volvulus* and more investigation of the cuticle of the homologous worm *Caenorhabditis elegans*. Collagens are among the most abundant components of the *C. elegans* cuticle, help to maintain worm structure, and may help to resist a variety of stresses including infection and toxins.^43^ With both methods of microscopy used in this study, we observed what appeared to be a banding pattern on the Mf surface staining with anti-OVOC12404 antibodies. Previous research has demonstrated that both *O. volvulus* adult females and Mf have annular ridges in their cuticle.^44^^,^^45^ Also, a similar localization pattern was previously reported for a different *O. volvulus* cuticular collagen, Ovcol-1.^11^ Therefore, we think this localization and banding pattern are further evidence that OVOC12404 is a component of the cuticle of the Mf. Given its location on the surface of the Mf, we predicted that the host would have opportunity to develop antibodies to this antigen.

The previously discovered *O. volvulus* collagen Ovcol-1 has a similar localization pattern to what we describe here and 53% sequence identity to OVOC12404.^11^ This sequence similarity is based on the glycine residues in the Gly-X-Y tripeptide repeat that make the collagen triple helix, as well as conserved amino acids within the amino–terminal signal sequence. Based on its smaller amino-terminal, non-triple helix domain and its uninterrupted triple helix domain, we propose that OVOC12404 is in the same collagen family as the *O. volvulus* collagens Ovcol-1 and OVOC12544 (collagen-like protein), the previously named “mini-chain collagens.”^11^ As no putatively immune individuals were evaluated in this study, it is challenging to directly compare the sensitivity and specificity of the antibody responses to these two filarial collagens.

The serology test we developed detects IgG antibodies to OVOC12404. Traditionally, filarial serodiagnostic tools have relied on identification of IgG4 to improve specificity. However, we found that the total IgG assay for OVOC12404 provided higher sensitivity than the IgG4 assay without sacrificing specificity. Interestingly, several other proposed onchocerciasis biomarkers including Ovcol-1, OvMANE1, and the peptides OvMP-1, OvMP-23, and OvNMP48 also had improved results with antibody subtypes other than IgG4.^11^^–^^13^

The sensitivity of our anti-OVOC12404 assay was 76.5% based on results obtained with banked clinical samples from both Ghana and Uganda. This exceeds the WHO TPP goal of ≥60% for mapping but is lower than the MDA stopping decision sensitivity goal of ≥89%. We did find variable sensitivity with samples from the two countries. This could be because of numerous factors, including different local onchocerciasis infection intensities and differences in prior MDA in the areas where samples were collected. For example, some of the samples collected in Ghana were from people who had been treated previously, although none had been treated with ivermectin in the 6 months before the study began.^19^^,^^46^ Although ELISA values were not significantly correlated with skin-snip Mf densities, the samples from Uganda were less likely to have antibodies to OVOC12404 but had significantly higher Mf densities than patients in Ghana who donated plasma. Prior studies have found an inverse relationship between skin-snip Mf counts and anti-Ov16 antibody levels. It is possible that antibodies to OVOC12404 were lower in Uganda because of antigen excess, or absorption of antibodies by Mf in the skin. It is also possible that anti-OVOC12404 antibodies are involved in immunity to the Mf stage and that their presence helps lower Mf counts. Further studies are needed to clarify the relationship between Mf and anti-OVOC12404 antibody abundance and to assess the sensitivity of this assay in different epidemiological settings.

Assessment of specificity of this assay is complicated by the difficulty in obtaining samples from people infected with *Loa loa *or *Mansonella perstans* who have no potential for exposure to onchocerciasis. The major limitation of this study is the availability of appropriate filarial samples to assess specificity. The specificities for those samples were lower than our goal, but it is unclear if the positive samples are truly from cross-reactive antibodies or because of co-endemicity of onchocerciasis with other filarial parasites in the study areas. This is clearly demonstrated with our results from people with lymphatic filariasis, where samples from areas without co-endemic onchocerciasis had an assay specificity of 97.6%, which is close to the TPP goal of 99.8%, whereas samples from areas with co-endemic onchocerciasis had a specificity of only 57%. Other filarial collagens could contribute to cross reactivity. However, given the relatively low sequence similarity between OVOC12404 and other filarial collagens, we propose that the apparent cross reactivity observed in this study is because of *O. volvulus* exposure. We are searching for other sera sets that could help clarify the diagnostic specificity of this assay.

Our study of pre- and post-treatment plasma samples showed that antibodies to OVOC12404 significantly decreased after treatment. IgG4 antibodies to Ov16 cannot differentiate between current and past infections.^5^^,^^6^ We directly compared the antibody levels to OVOC12404 and Ov16 by ELISA using the same pre- and posttreatment plasma samples. The OVOC12404 antibody assay was slightly more sensitive than the Ov16 antibody assay for the pretreatment samples, although the rates of positive samples were not significantly different. However, in the sera set used in this study, anti-OVOC12404 antibodies declined after treatment, whereas anti-Ov16 antibody levels were essentially unchanged. Further testing with other sample sets would be helpful. However, based on our results, it appears that the OVOC12404 antibody assay may have advantages over other antibody tests for identifying *O. volvulus* infections after onchocerciasis control efforts.

We envision that an OVOC12404 serodiagnostic test could be useful for several scenarios. For example, a combination assay that detects either OVOC12404 or Ov16 would provide high sensitivity. However, detection of OVOC12404 antibodies alone might be a better tool than Ov16 antibodies for assessing the impact of interventions and for endpoint assessments.

Further analysis is needed to characterize the sensitivity and specificity of this assay with fresh biological samples and to determine how long after treatment anti-OVOC12404 antibodies remain in circulation. Importantly, this study used an interesting approach to identify a new diagnostic antigen for onchocerciasis serology. This antigen has the potential to complement serodiagnostic tests based on Ov16 and thereby aid in the campaign to eliminate human onchocerciasis.

## Supplemental Materials

10.4269/ajtmh.24-0793 Supplemental Materials
