# Supplementary material for: An Antibody Biomarker Associated with Onchocerca volvulus Microfilariae Identified by Proteomic Analysis of Parasite Tissues Isolated from Paraffin Embedded O. volvulus Nodules by Laser Capture Microdissection
Source: Am J Trop Med Hyg. 2025 Jun 3;113(2):324–33. doi: 10.4269/ajtmh.24-0793 (PMC12403327; doi:10.4269/ajtmh.24-0793)
Supplement: Supplemental Materials [file tpmd240793.SD2.pdf]

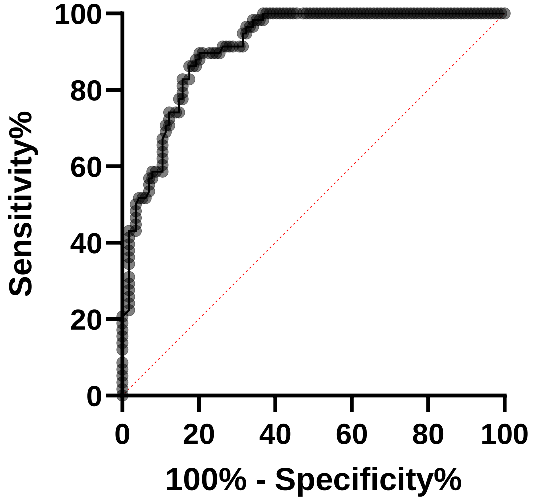

Fig S1. Receiver operating curve analysis of anti-OVOC12404 IgG ELISA results. All data from patients with onchocerciasis were included as the patient data. For this analysis, the control samples included data from people with lymphatic filariasis (outside onchocerciasis endemic areas) and the non-endemic controls. The area under the curve was 0.9117. This resulted in an assay sensitivity of 63% and specificity of 98%.

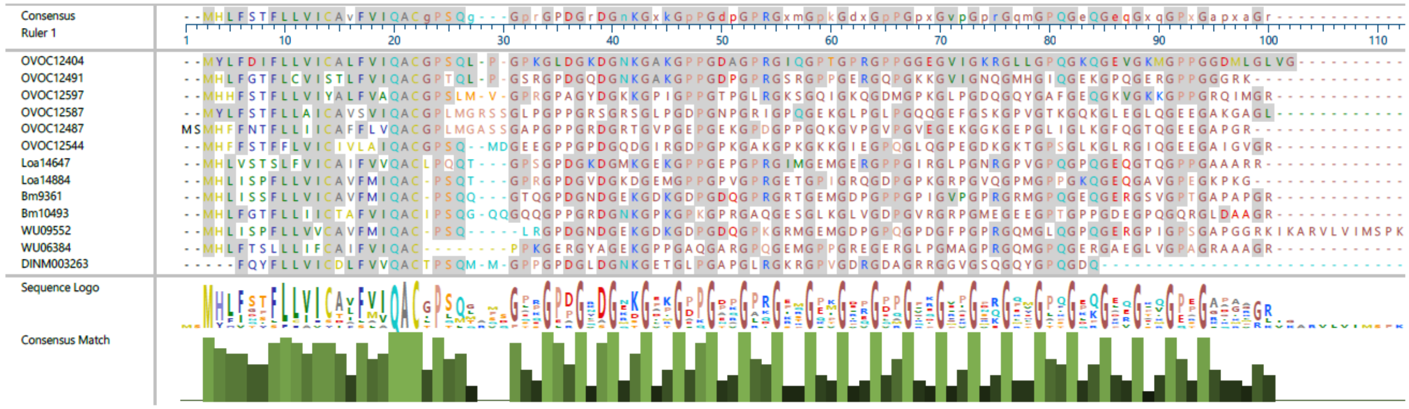

Fig S2: Amino acid alignment of filarial proteins with a collagen triple helix domain. Amino acid text color is based on the Shapely amino acid color scheme. Each amino acid is shaded in grey if it is conserved with OVCO12404. Protein percent identities relative to OVOC12404 are listed in parentheses below. The figure includes proteins from *O. volvulus*: OVOC12404 (100%), OVOC12491 (68%), OVOC12597, also known as Ovcol-1, (53%), OVOC12587 (50%), OVOC12487 (47%), and OVOC12544, also known as collagen like protein, (46%); from *W. bancrofti*: WU09552 (57%) and WU06384 (53%); from *B. malayi*: Bm9361 (65%) and Bm10493 (53%); from *L. loa*: Loa14647 (65%) and Loa14884 (59%); and from *D. immitis*: DINM003263 (60%).

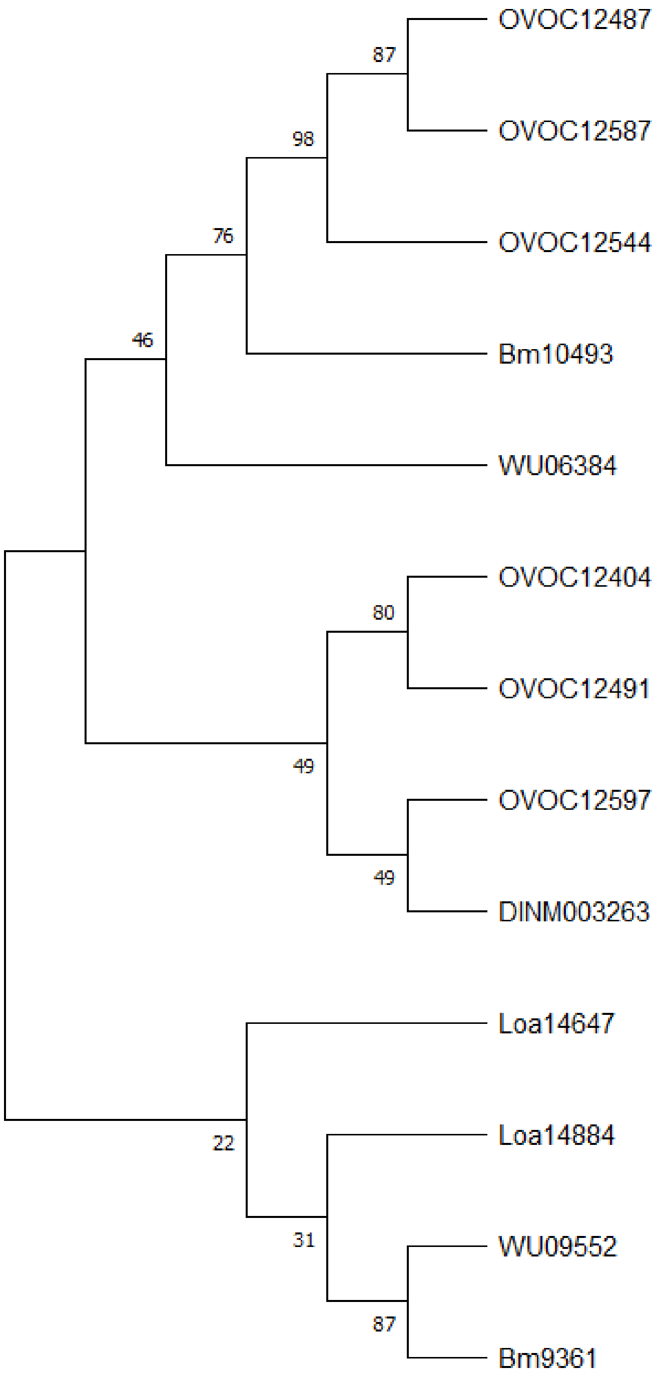

Fig S3: Phylogenetic tree of filarial collagen proteins. The figure demonstrates the bootstrap consensus Maximum Likelihood phylogenetic tree of the collagen triple helix containing proteins from *O. volvulus*, *W. bancrofti*, *B. malayi*, *L. loa*, and *D. immitis* that were listed in Fig S1. The tree was inferred from 1000 replicates. The number at each branchpoint indicates the percent of trees where the named proteins clustered together.

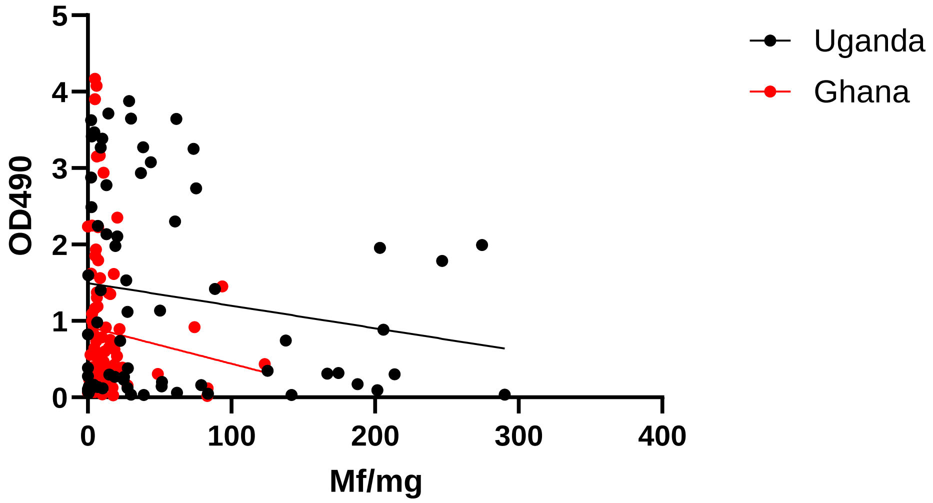

Fig S4. Anti-OVOC12404 IgG ELISA results do not correlate with microfilarial density. Graph shows the OD_{490} values for the anti-OVOC12404 IgG ELISA plotted against that individual's Mf density as determined by skin snip. The color of the data point indicates the country in which the sample was obtained. The OD_{490} values do not correlate with Mf density in either country, based on Spearman R values, although there was a trend towards higher OD_{490} values in people with lower Mf densities.

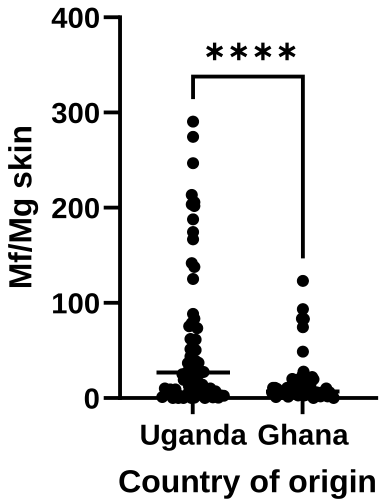

Fig S5. Mf densities from Uganda and Ghana. The Mf counts in Mf per mg of skin snip from people that donated plasma samples used in the study. Mf counts are plotted based on country of origin. These counts are significantly different by Mann-Whitney ($p<0.001$).
